# Hypertension and prehypertension among adolescents in secondary schools in Enugu, South East Nigeria

**DOI:** 10.1186/1824-7288-39-70

**Published:** 2013-11-02

**Authors:** Fortune A Ujunwa, Anthony N Ikefuna, Ada RC Nwokocha, Josephat M Chinawa

**Affiliations:** 1 Department of Paediatrics, University of Nigeria Teaching Hospital, Enugu 400001, Nigeria

**Keywords:** Adolescent, Hypertension, Prehypertension

## Abstract

**Background:**

Hypertension is a prevalent cardiovascular disease risk factor among blacks and adolescent hypertension can progress into adulthood.

**Objective:**

To determine the prevalence of hypertension and prehypertension among secondary school adolescents in Enugu South East Nigeria.

**Methodology:**

A study of 2694 adolescents aged 10-18 years in Enugu metropolis was carried out. Socio-demographic profile anthropometric and blood pressure readings were obtained. Derived measurements such as Prehypertension, hypertension and BMI were obtained.

**Results:**

The results showed that the mean systolic blood pressure and diastolic blood pressure for males were 106.66+ 11.80 mmHg and 70.25 + 7.34 mmHg respectively. The mean SBP and DBP for females were 109.83+ 11.66 mmHg and 72.23 + 8.26 mmHg respectively (p < 0.01). Blood pressure was found to increase with age. Prevalence of hypertension and prehypertension was 5.4% and 17.3% respectively with a higher rate in females (6.9%) than males (3.8%). Prevalence of prehypertension among males and females were 14.3% and 20.1% respectively. The prevalence of obesity was 1.9%.

**Conclusion:**

Modifiable risk factors exist among adolescents. Early lifestyle modification and a strengthened school health are recommended.

## Introduction

Hypertension is the commonest non communicable disease affecting both sexes in all races [1]. It is the most prevalent cardiovascular disease risk factor worldwide. It has been shown that hypertension seen in children can progress into adulthood thus contributing to the increase in the cardiovascular morbidity and mortality in adults [2-4].

Many studies [5-7] on hypertension worldwide have been on middle aged and elderly patients giving the impression that hypertension is a disease of those age groups. The astonishment and disbelief with which young individuals react to the diagnosis of hypertension is a pointer to this assertion [7]. Even among clinicians who take care of children the disease is highly under diagnosed [8].

The risk of developing hypertensive cardiovascular complications is greater in younger than in older individuals [9]. The younger the age of onset of hypertension the greater the reduction in life expectancy if the blood pressure is left untreated [10]. It has also been noted that even asymptomatic adolescents with mild blood pressure elevations can have target organ damage [2,3]. Adolescents with high blood pressure have a significantly greater clustering effect of metabolic syndrome factors when compared to adolescents with low blood pressure [11]. Mortality and disability adjusted life years lost due to hypertension related diseases have been noted to be increasing especially in the developing countries of which the associated health problems and cost is a cause of grave concern [12]. It is important from the disease prevention stand point to consider elevated blood pressure as a risk factor in the Paediatrics age group well before clinical manifestation of the disease become apparent in them or later in life [11]. Identifying children and adolescents at risk is the first step in preventing the disease and its risk factors which include cigarette smoking alcohol intake physical inactivity, obesity, steroid abuse family history of hypertension, low birth weight hypercholesterolemia, hyperinsulinaemia, homocystinaemia and poor nutrition [13]. These risk factors have been found to be prevalent among the adolescent age group, and present serious challenge in intervention since they are usually difficult to change [14,15].

The study was designed to determine the prevalence of arterial hypertension and pre-hypertension among secondary school adolescents in Enugu and also to estimate the proportion of some modifiable risk factors for arterial hypertension; such as obesity, cigarette smoking and alcohol consumption among the adolescents.

## Methodology

The study was a cross sectional survey of 2694 secondary school adolescents aged 10-18 years. The study was conducted from October 2009 to June 2010. A multistage sampling method was used to select the subjects from the schools in the three local government areas that make up the metropolis. Schools were stratified into all boys’, all girls’ and co-educational schools. Ethical clearance was obtained from the University of Nigeria Teaching Hospital ethics and Research committee. The participants were apparently healthy secondary school adolescents subjects. Those who were preadolescents were excluded as well as subjects who volunteered history of chronic illness such as bronchial asthma, renal pathologies such as nephrotic syndrome and on chronic medication were also excluded. Data was collected from the participants using a pretested questionnaire. Anthropometric measurements were done using stadiometer. Obesity and overweight were defined as body mass index greater than or equal to age specified international cut off points for child overweight and obesity, [16] while thinness(under-weight) was defined using the international cutoff points established by Cole et al. [17]. Blood pressure was measured according to American heart association recommendations on each occasion using mercury sphygmomanometer [17]. The first and fifth korotkoff sounds were used as the systolic and diastolic blood pressures respectively [18]. Three readings were taken on each occasion and the average of the three readings taken as the blood pressure. Hypertension was defined as blood pressure above 95^th^ percentile of the blood pressure for age, height and sex while pre-hypertension was defined as blood pressure above the 90^th^ percentile but below the 95^th^ percentile or greater than or equal to 120/80 mmHg compared with the Working Group on blood pressure standards recommendations [19]. Those that had elevated blood pressure at first visit had two other blood pressure measurements on different occasions in the same venue with at least a week interval between measurements and high blood pressure was staged according to working group recommendations [19]. Adolescents with significant alcohol intake was classified as those that consume more than 16 g of alcohol per day while those that had insignificant alcohol intake was classified as those that consume less than 8 g per day [20]. Smokers were classified as those who smoke any form of tobacco or marijuana either occasionally or daily.

Data were analyzed with computerized statistical package for social sciences (SPSS) version 15.0. Data base was created using the soft ware and frequency of variables obtained. Students’ t- test was used to compare mean systolic and diastolic blood pressures and other numeric variables. Chi-squared test was used to compare categorical variables. Correlation coefficient was used to describe strength of association between variables. Significant probability value was P < 0.05.

## Results

A total of 2694 subjects participated in the study, this comprised 1293 males and 1401 females; male to female ratio of 1:1.08. The mean age of the population was 15.03 ± 1.89 years (males 14.86 ± 1.97 years, and females 15.18 ± 1.80 years). The age and sex distribution of the respondents is shown as Table 1.

**Table 1:** Age and sex distribution of the study population

**Age group (years)**	**Male n (%)**	**Female n (%)**	**Total n (%)**
10 < 11	11 (0.9)	11 (0.8)	22 (0.8)
11 < 12	51 (3.9)	36 (2.6)	87 (3.2)
12 < 13	117 (9.1)	84 (6.0)	201 (7.5)
13 < 14	148 (11.5)	121 (8.6)	269 (10.0)
14 < 15	229 (17.7)	188 (13.4)	417 (15.5)
15 < 16	201 (15.5)	277 (19.8)	478 (17.7)
16 < 17	235 (18.2)	352 (25.1)	587 (21.8)
17 < 18	179 (13.8)	208 (14.8)	387 (14.4)
18 < 19	122 (9.4)	124 (8.9)	246 (9.1)
Total	1293 (100)	1401 (100)	2694 (100)

### Anthropometry

The mean height of males and female adolescents were 1.63 ± 0.11 meters and 1.60 ± 0.06 meters p < 0.01. The mean weight for females was 54.44 ± 9.18 Kg and for males 52.96 ± 12.19 Kg p < 0.01. Both the weight and the height increased linearly with age of the subjects. The mean waist and hip circumference of female adolescents were 71.44 ± 7.19 cm and 84.15 ± 23.25 cm respectively while the mean waist and hip circumference for males were 69.22 ± 24.45 cm and 79.14 ± 8.64 cm respectively p < 0.01. The mean body mass index of males and females were 19.81 ± 3.61 Kg/M^2^ and 21.16 ± 3.29 Kg/M^2^ respectively. The anthropometric variables were found to increase with age (Table 2) and there were statistically significant gender difference in the mean differences of the anthropometric variables Table 3. There was also positive correlation between the anthropometric variables and blood pressure pattern.

**Table 2:** Age distribution of anthropometric variables

**Age (years)**	**Weight (kg) ± SD**	**Height (m) ± SD**	**BMI (kg/m**^**2**^**) ± SD**	**Hip circ (cm) ± SD**	**Waist circ (cm) ± SD**
10 < 11	51.03 ± 11.51	1.53 ± 0.05	21.67 ± 4.40	84.05 ± 9.88	74.75 ± 11.24
11 < 12	42.45 ± 9.58	1.51 ± 0.06	18.41 ± 3.17	73.78 ± 8.94	65.85 ± 8.28
12 < 13	43.43 ± 9.58	1.53 ± 0.07	18.23 ± 3.55	74.25 ± 8.80	68.66 ± 4.27
13 < 14	46.75 ± 9.48	1.56 ± 0.07	19.19 ± 3.42	77.38 ± 8.61	67.35 ± 7.22
14 < 15	49.57 ± 8.84	1.58 ± 0.08	19.39 ± 3.35	79.55 ± 7.05	69.45 ± 30.07
15 < 16	54.20 ± 8.20	1.63 ± 0.07	21.44 ± 2.68	83.07 ± 7.66	70.35 ± 7.15
16 < 17	57.47 ± 9.40	1.65 ± 0.08	21.21 ± 3.40	84.85 ± 6.76	71.50 ± 5.96
17 < 18	59.76 ± 8.79	1.66 ± 0.08	21.54 ± 3.55	87.20 ± 42.70	72.32 ± 6.62
18 < 19	61.72 ± 9.62	1.67 ± 0.09	21.99 ± 3.53	85.26 ± 6.60	72.41 ± 6.81
Mean	53.73 ± 10.76	1.61 ± 0.09	20.40 ± 3.51	82.18 ± 18.08	70.40 ± 17.79
f (ANOVA)	138.17	117.56	36.58	17.83	3.53
P	0.001*	0.001*	0.001*	0.001*	0.001*

*Statistically Significant p- value at 0.05 level of significance;

Circ – circumference. BMI-Body Mass Index.

**Table 3:** Mean characteristics of the study population

**Variables**	**Male**	**Female**	**T**	**P**
Age	15.03 ± 1.97 years	14.86 ± 1.89 years	4.52	0.01*
Weight	54.44 ± 9.18 Kg	52.96 ± 12.19 Kg	3.76	0.01*
Height	1.63 ± 0.11 M	1.60 ± 0.06 M	−6.69	0.01*
waist circumference	71.44 ± 7.19 cm	84.15 ± 23.25 cm	3.13	0.01*
hip circumference	69.22 ± 24.45 cm	79.14 ± 8.65 cm	1.79	0.06
BMI	19.81 ± 3.61 Kg/M^2^	21.16 ± 3.29 Kg/M^2^	11.51	0.01*

*Statistically significant p-values at 0.05 level of significance.

### Blood pressure pattern

The mean systolic and diastolic blood pressures of the population were 108.31 ± 11.83 mmHg and 71.21 ± 7.89 mmHg respectively. The mean SBP and DBP for males were 106.66 ± 11.80 mmHg and 70.25 ± 7.34 mmHg respectively while the mean SBP and DBP for females were 109.82 ± 11.66 mmHg and 72.23 ± 8.26 mmHg respectively. There was a statistically significant gender difference in the mean SBP and DBP of the study population.

In terms of adolescent age groups the mean SBP and DBP of males and females were significantly different at 10-13 years (early adolescent) and 14–16 years (mid adolescent) Table 4.

**Table 4:** Distribution of mean blood pressures of the study population according to sex and age group

**Age group (years)**	**SBP (mmHg)**		**t**	**p**	**DBP (mmHg)**		**t**	**p**
	**Male**	**Female**			**Male**	**Female**		
10–13	99.43 ± 10.45	104.73 ± 11.69	5.74	0.01*	66.85 ± 6.51	69.26 ± 8.31	3.89	0.01*
14–16	107.30 ± 11.12	109.94 ± 11.06	4.552	0.01*	70.46 ± 7.00	72.25 ± 7.97	4.54	0.01*
17–18	113.11 ± 10.40	113.40 ± 11.69	0.340	0.73	73.45 ± 7.37	74.41 ± 8.27	1.53	0.13

*Statistically significant p-values at 0.05 level of significance.

**Figure 1:**
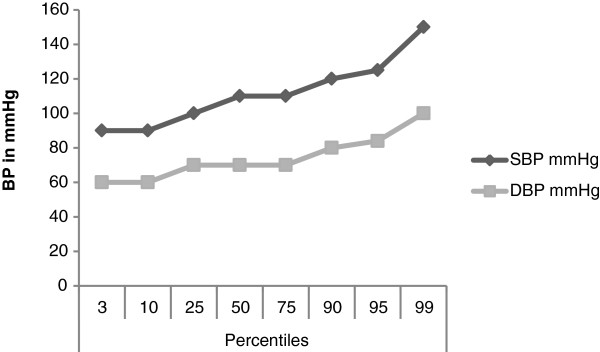
Percentiles of the systolic and diastolic blood pressure of the study population.

Blood pressure was also shown to increase with age. The average yearly increase for DBP was 1 mmHg while the average annual increase of SBP was 2 mmHg. The study group 50^th^ percentile for both the SBP and DBP were 110 mmHg and 70 mmHg respectively while the 95^th^ percentile value was 125 mmHg for SBP and 84 mmHg for DBP. See Figure 1.

Of the 2694 study respondents the 322 (12%) were found to have hypertension, 364 (13.5%) prehypertension and 2008 (74.5%) had normal blood pressure after the first set of measurements.

In the last series of measurements, it was found that there were 146 adolescents that had hypertension giving a prevalence of 5.4%. Among these 97 (6.9%) were females and 49 (3.8%) were males. Among the hypertensives 99 subjects (67.8%) were classified as stage I while 47 subjects (32.2%) had stage II hypertension. About 77.3% of the subjects had normal blood pressure values while 467 (17.3%) had pre-hypertension. The prevalence rate of pre-hypertension in males and females were 14.3% and 20.01% respectively p < 0.01.

Tables 5, 6 and 7 illustrate the distribution of Blood pressure pattern according to gender, body Mass Index and obesity.

**Table 5:** Distribution of blood pressure profile according to gender

**Blood pressure profile**	**Male n (%)**	**Female n (%)**	**Total (%)**	**χ**^**2**^	**p-value**
Normal BP	1059 (81.9)	1022 (73.0)	2081 (77.3)	0.44	0.51
Pre-hypertension	185 (14.3)	282 (20.1)	467 (17.3)	13.50	0.01*
Hypertension	49 (3.8)	97 (6.9)	146 (5.4)	10.65	0.01*
	1293 (100)	1401 (100)	2694 (100)	2.89	0.09

*Statistically significant p-values at 0.05 level of significance.

**Table 6:** Distribution of blood pressure pattern according to body mass index

**BMI**	**Normal (%)**	**Prehypertension (%)**	**Hypertension (%)**		**χ**^**2**^	**p-value**
Underweight	177 (6.6)	26 (1.0)	16 (0.6)	219 (8.1)	222.6	0.01
Normal	1723 (64)	326 (12.1)	87 (3.2)	2136 (79.3)	2193.5	0.01
Overweight	163 (6.1)	98 (3.6)	26 (1.0)	287 (10.7)	98.2	0.01
Obese	18 (0.7)	17 (0.6)	17 (0.6)	52 (1.9)	0.038	0.98
Total	2081 (77.3)	467 (17.3)	146 (5.4)	2694 (100)	2395.1	0.01

**Table 7:** Blood pressure profile and obesity

**Blood pressure**	**Non-obese n (%)**	**Obese n (%)**	**Total n (%)**	**χ**^**2**^	**p-value**
Normal BP	2063 (99.13)	18 (0.87)	2081 (100)	36.65	0.01
Pre hypertension	450 (96.36)	17 (3.64)	467 (100)	3.92	0.04
Hypertension	129 (88.36)	17 (11.64)	146 (100)	62.41	0.01
Total	2642 (98.1)	52 (1.9)	2694 (100)		

### Blood pressure and modifiable risk factors

It was found that 219 (8.1%) of the respondents were underweight this comprised 117 (53.4%) females and 102 (46.6%) males p = 0.31, 287 (10.7%) were overweight 186 (64.8%) females and 101 (35.2%) males p < 0.01. The prevalence of hypertension and prehypertension among the overweight subjects were 9.1% and 34.1% respectively.

Obesity was found in 52 subjects of the study population giving a prevalence rate of 1.9%. Of the 52 obese subjects there were 33 (63.5%) females and 19 (36.5%) males. Normal blood pressure readings was found in 18 (34.6%) of the obese subjects while prehypertension and hypertension occurred in 17 (32.7%) and 17 subjects (32.7%) respectively.

Alcohol consumption was noted among 1035 (38.4%) of the study population. Significant daily intake of alcohol was noted among 2.2% of those that consumed alcohol while 92.3% had non significant intake. Alcohol intake was noted more among the males than the females (611 males and 424 females p < 0.01). However blood pressure profile was independent of alcohol intake. It was also observed that 61 respondents smoked cigarette while 8 respondents smoked marijuana, this comprised 58 males and 11 females giving a prevalence rate of 2.6% blood pressure profile was also independent of the smoking attitude of the adolescents.

## Discussions

The prevalence of hypertension in the study population was 5.4% with male and female prevalence rates of 3.8% and 6.9% respectively. This difference in the observed prevalence of hypertension may probably be due to a greater delay in boys in completing pubertal development and attaining final height than in females. In addition greater body mass index and waist circumference noted in females may have contributed to this difference in blood pressure. The observed prevalence rates shows an increase in the prevalence rate of hypertension when compared to a rate of 3.3% obtained in Southwest of Nigeria [21] though lower than values documented by Ejike et al. [22] in Kogi as well as Mijinyawa in Kano [23]. The difference in prevalence rates may be due to varying methodology, different criteria for diagnosis of hypertension and regional variations. Nevertheless the observed prevalence rate falls within the documented prevalence rate of adolescent hypertension of 1-13% in Nigerian adolescents [21-25]. The implication of this is that hypertension can be detected in the adolescent age group. This calls for regular screening of this group of individuals who hitherto were assumed to be a healthy population. This will help in early detection of this disease condition as well as initiate early treatment for those affected in order to avoid target organ damage due to untreated hypertension.

The detection of individuals who have blood pressure greater than or equal to 95^th^ percentile for age will assist in the proper evaluation and classification of these individuals, because elevated blood pressure may be an early pointer to some chronic diseases such as nephritic syndrome, chronic kidney disease, adrenal and renal tumors hypercholesterolemia among others [26]. It is possible that some of the adolescents identified to have hypertension in this study may be prone to these diseases. Thus there is need for further studies and evaluation of any adolescent found to have hypertension by assessing for clinical and laboratory features of some of these diseases.

The observed prevalence of pre-hypertension was lower than values obtained by Ejike et al. [22] in North Central Nigeria, however higher prevalence of prehypertension noted in females was also documented by previous authors [27,28]. This group of individuals in the pre-hypertensive range are those who might become hypertensive later in life if adequate life style modifications such as weight reduction, regular exercise, reduction of alcohol intake and smoking are not instituted thereby increasing the prevalence of hypertension and its attendant complications.

In the present study the mean SBP and DBP increased with ages in both sexes. There was a more rapid increase between the ages of 13 and 15 years which corresponds to the mid adolescent age group. This age group is usually the period of puberty in adolescents. Similar finding has been documented in the earlier studies [22,29]. The increase noted in the mid adolescent period may be attributed to rapid hormonal changes and increase in body size associated with pubertal growth. The average yearly increase of 1 mmHg for DBP and 2 mmHg for SBP noted in the study is similar to the values observed in Jordanian children [30].

Blood pressure pattern has been documented to differ between male and females. Both the mean SBP and DBP were significantly higher in females than in males especially during the early and mid adolescent stages. A similar trend has also been noted by Jaddou et al. [30] and Ayoola [31]. This gender difference in blood pressure pattern may be attributed to hormonal changes that occur during puberty which has been noted to occur more rapidly in females than in males. The psychosocial stress associated with menarche has also been documented to cause an increase in blood pressure in early and mid adolescent stage [32].

Moreover females were noted to have higher body weight and body mass index than males in the study population. It is possible that these differences may have contributed to the observed differences noted in the blood pressure pattern of both sexes. The anthropometric variables were shown to correlate positively with age, SBP and DBP, and this is consistent with the report of several authors [21,22,30]. The implication of this increase in blood pressure with age is that it may continue into the adult stage hence further increasing the prevalence of hypertension in the adult group with its attendant morbidity disability and mortality.

Obesity has been noted to be associated with hypertension, the observed prevalence in the present study is similar to earlier studies in developing countries [33,34] but lower than values obtained from the United States of America [35]. The prevalence rate of 36.45% for obesity related hypertension is high for a developing economy that is already battling with infectious disease burden hence the need for urgent prevention strategies.

Blood pressure pattern was independent of risk factors such as smoking and alcohol intake of the participants in this study, this may be due to the quantity consumed and duration of consumption which is usually longer in adult population. It is possible that these young smokers and consumers of alcohol may continue up to adulthood as had been documented by earlier authors [36,37]. Thereby predisposing them to the long term effect of these risk factors hence the need for effective health education, lifestyle modification and counseling for these adolescents who are already exposed to these products in order to effect attitudinal change and prevent future complications such as hypertension.

The limitations noted in the study include exclusion of subjects that was based on volunteered information and biochemical analysis that were not done. These could have helped in further identifying subjects with possible secondary hypertension also the lipid profile and presence of insulin resistance were not studied, this leaves room for further studies in these areas. The timing of feeding of the participants were not controlled in the study this could have controlled for transient blood pressure variability immediately after feeding.

Our study revealed a relatively high prevalence rate of hypertension and prehypertension among adolescents studied and these were more common among female subjects.

It is therefore recommended that periodic screening and monitoring of blood pressure of adolescents should be incorporated into the school health programme, while general public health education on hypertension and its associated risk factors should be strengthened.

## Consent

Written informed consent was obtained from the patients for the publication of this report and any accompanying images.

## Competing interests

The authors declare no competing interests.

## Authors’ contributions

FAU was involved in conceptualizing and writing of the manuscript. ANI participated in the sequence alignment and drafting of the manuscript. ARCW contributed in the proof reading and correction of the manuscript while JMC Helped in editing and rewriting the manuscript to journal requirements. All authors contributed to the conception, writing and proof reading of this manuscript.
